# Spinal muscular atrophy in the disease-modifying therapy era: successes, limitations and future directions

**DOI:** 10.3389/fmmed.2026.1901311

**Published:** 2026-08-25

**Authors:** Madison M. Sexton, Emma W. Crow, Congyue Annie Peng

**Affiliations:** 1 Department of Bioengineering, Clemson University, Clemson, SC, United States; 2 Department of Genetics and Biochemistry, Clemson University, Clemson, SC, United States

**Keywords:** atrophy, disease-modifying therapies, neuron, SMN, therapy

## Abstract

Spinal Muscular Atrophy (SMA) is a rare and debilitating neurodegenerative disease characterized by the progressive loss of motor neurons in the spinal cord, leading to muscle weakness, respiratory failure, and premature mortality. The pathogenesis of SMA is highly complex and the investigation of downstream pathways and specific cellular mechanisms is still ongoing. In recent years, three FDA-approved disease-modifying therapies, nusinersen, risdiplam, and onasemnogene abeparvovec, have improved the quality of life for patients with SMA and have eased the management of associated symptoms. However, unmet needs remain as comorbidities become increasingly apparent in the era of disease-modifying therapies. Despite the remarkable progress achieved over the past decade, continued research is essential to further improve the quality of life, clinical outcomes, and standard of care for individuals living with SMA.

## 1 Introduction

Spinal Muscular Atrophy (SMA) is an inherited neurodegenerative disease caused by the deletion of the Survival Motor Neuron 1 (*SMN1*) gene. Reduced expression of the SMN protein causes atrophy of motor neurons (MN) within the anterior horn of the spinal cord (Lefebvre et al., 1995). It is the leading cause of hereditary infant mortality (Darras et al., 2015), with an incidence of 10 in 100,000 (Verhaart et al., 2017). Recent developments of disease-modifying therapies (DMTs) have changed the landscape of SMA treatment within the past decade (Reilly et al., 2023). However, unmet needs remain. In 2024, approximately 75% of patients in the CureSMA database had received at least one DMT, but only 47% of children and 19% of adults living with SMA are ambulatory (Cure, Cure SMA, 2025). In the post-DMT era, it will be essential to prioritize improving patient quality of life, whether through optimizing current DMTs or developing new treatments to work synergistically with them.

In this review, we detail the discovery of the disease-causing gene and the classification of the disease phenotype. We also explore SMN’s role in RNA metabolism and discuss potential mechanisms of motor neuron-specific disease manifestations. Finally, we highlight pivotal clinical trials from the DMTs and review alternative routes for new treatment strategies.

## 2 Discovery, classification and clinical features of SMA

SMA was first documented in 1891 by neurologist Guido Werdnig when he made a case report of two brothers who had progressive muscular atrophy. For 104 years following Werdnig’s discovery, many different clinical manifestations of SMA had been reported, leading to the assumption that genetic heterogeneity was at play (Nishio et al., 2023). However, in 1990, all forms of the disease had been mapped to chromosome 5q (Gilliam et al., 1990). It was not until 1995 that the landmark paper by Lefebvre et al. identified the SMA disease-causing gene. Previous studies identified the 5q13 locus as a region of interest for the disease-causing gene through linkage analysis, and polymorphic DNA markers from yeast artificial chromosomes were identified. Through the genetic and physical mapping of this locus, the authors identified the causative gene, ^T^BCD541 and its paralog, termed ^C^BCD541. The telomeric copy was aptly named survival motor neuron 1 (*SMN1*), and the centromeric copy was named *SMN2*. They found that *SMN1* was deleted in 98.7% of patients and mutated in 1.3% within a 229-patient cohort (Lefebvre et al., 1995).

*SMN1* and *SMN2* are around 20 kb, contain nine exons, and are virtually homologous except for a singular base pair transition from a C to T in exon seven of *SMN2* (Monani et al., 1999). This both inactivates an exonic splicing enhancer and introduces an exonic splicing silencer (Lorson et al., 1999), (Lorson and Androphy, 2000). As a result, around 90% of the protein originating from *SMN2* is truncated and degraded by cell machinery. The duplication of *SMN2* is evolutionarily recent and is unique to *Homo sapiens* (Rochette et al., 2001). The severity of SMA is inversely related to the number of *SMN2* copies a patient has. Chronic deficiency of the SMN protein causes MN degeneration within the anterior horn of the spinal cord. It is not known why MNs are specifically afflicted. As MNs progressively atrophy, they recede from the surrounding muscle, making it difficult for the neurons to connect directly with the muscle (Ottoboni et al., 2026).

**FIGURE 1:**
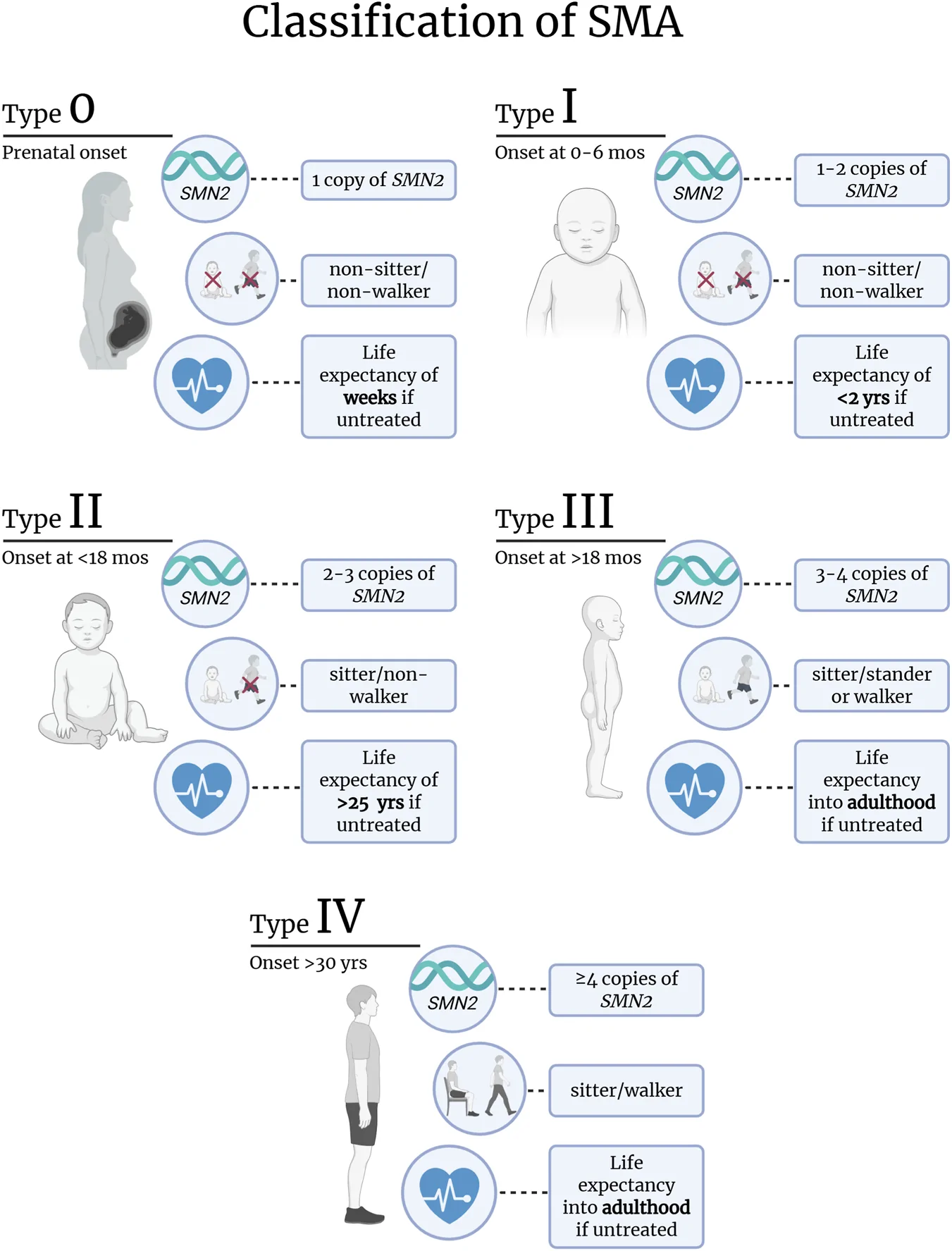
Classification of SMA. Types 0-IV SMA are distinguished by copy number of *SMN2* and onset of disease. Early onset of SMA is associated with limited motor ability, and a significantly shortened lifespan if the patient is untreated. Created in BioRender. Peng, C. (2026) https://BioRender.com/k1apo80.

Currently, there are five types of SMA, distinguished by major motor milestones and copy number of *SMN2* (Figure 1). The most common form of SMA, classified as Type I, has an infantile onset, with motor deficits beginning within 6 months of age on average. Motor ability steadily declines until death at around 2 years of age if untreated. Type 0 SMA is the most severe, with onset beginning in the womb and severe hypotonia and respiratory insufficiency beginning almost immediately upon birth (Al Dakhoul, 2017; Singh et al., 2018; Arnold et al., 2015).

### 2.1 Comorbidities of SMA

Although the degenerative neuromuscular phenotype is the primary concern for patient care, many multi-system effects significantly impact a patient’s quality of life. With the recent availability of DMT’s, patients are becoming stronger and more likely to live longer. However, this introduces new hurdles for patients, researchers, and physicians to overcome as comorbidities become more apparent. In a recent case-control study, 71.4% of patients (25/35) reported experiencing comorbidities. They found that one comorbidity did not affect quality of life, but having more than one did (Blauciak et al., 2024).

#### 2.1.1 Orthopedic

Orthopedic abnormalities are a significant concern. 60%–90% of children with SMA have scoliosis, which can make it difficult to reach the spine for treatments requiring intrathecal injections like nusinersen. Untreated spinal curvature can lead to chest deformities, significantly impacting pulmonary function. Consequently, respiratory failure is the leading cause of mortality for SMA Types I and II (Prior et al., 2000). Prior to DMTs, spinal orthopedic care was often not considered in severe SMA patients due to their short survival. Rigid braces can be used for non-sitters; however, the treatment landscape is fluid as physicians and researchers determine the best route as patients live longer. Recommendations for treatment change as the spine matures, but generally a curvature of >50° angle is considered severe (Mercuri et al., 2018a). In children younger than 8–10 years old, instrumentation that allows continued spinal growth is recommended, like growing rods (McElroy et al., 2011) or magnetic bars (Yoon et al., 2014). For patients with a mature spine, spinal fusion surgery is the best option for most (Wang et al., 2007). Hip instability can also occur in SMA patients due to weakened pelvic muscles. Some argue against the need for surgery since hips are prone to partial dislocation even after surgery, but some insist that modern surgical techniques may be able to help those in severe pain (Vitale et al., 2022).

#### 2.1.2 Gastrointestinal

Gastrointestinal complications have also been documented as a common comorbidity to SMA, likely due to weakening of the abdominal and pelvic muscles (Wang et al., 2007). Caregivers of Type 1 patients have reported chronic constipation, dysphagia (Mercuri et al., 2018a), and gastroesophageal reflux complications (Prior et al., 2000). Difficulties with eating can result in metabolic abnormalities, including glucose intolerance and insulin resistance (Bowerman et al., 2012).

#### 2.1.3 Cardiopulmonary

Negative impacts on cardiopulmonary function are commonly observed in patients with SMA. Respiratory-related complications, as previously stated, are the leading cause of mortality in these individuals, with a recent review showing that the known cause of death in 72% of patients with SMA was related to respiratory failure (Finnegan et al., 2024). Multifactorial impacts on respiratory and pulmonary function include respiratory infections, restrictive lung disease, feeding-related respiratory complications, sleep-disordered breathing, and related impediments. Bulbar dysregulation and associated feeding difficulties can further impact pulmonary function and increase the risk of aspiration, often requiring noninvasive ventilation or additional nutritional support. Cardiac irregularities are less commonly observed but can be seen in infantile patients with Type 1 and 2 SMA. The most frequently reported abnormalities consisted of congenital heart defects in various regions, EKG abnormalities, and cardiomyopathy changes that vary with the type of SMA (Cui et al., 2022). Cardiopulmonary complications are a significant comorbidity and a leading cause of mortality in SMA patients, indicating further necessity for comprehensive respiratory and cardiac monitoring and management.

As the landscape of treatments for SMA patients continues to evolve, we must also focus on supportive care as patients live longer and comorbidities become more apparent. It should be a priority to strengthen the muscles that support peripheral organ function.

## 3 The SMN protein and potential mechanisms of disease

The SMN protein comprises 294 amino acids (38 kDa) and is ubiquitously expressed in all tissues of the body. It is essential for life, as SMN knockout in mice is embryonic lethal (Schrank et al., 1997). Expression of SMN is increased within the cerebral and spinal cord areas, especially within lower spinal cord MNs (Battaglia et al., 1997). A chronic deficiency of the SMN protein causes atrophy of these lower MNs, particularly within the anterior horn. It remains unclear why MNs are selectively affected, but it is likely due to their need for increased RNA metabolism to accommodate the complex, highly efficient signal-processing task.

The structure of the *SMN2* locus and the factors that interact with it heavily influence SMN protein stability and disease severity. *SMN1/2* is a highly repetitive region of the genome, which can make it difficult to sequence with next-generation sequencing technologies (Hall et al., 2026). However, several negative cis acting elements have been identified near the 5′ splice site of exon seven within *SMN2*, including a terminal loop structure 2, GC-rich sequence and an internal stem formed by a long-distance interaction. Increasing the accessibility of exon seven by interfering with these elements has shown to restore exon seven inclusion, leading to full length SMN protein (Ottesen, et al., 2023). Epigenetic mechanisms like methylation and histone acetylation have been shown to affect SMN expression and disease severity. The histone acetylation pattern of *SMN2* is distinct, with hyperacetylation found near the promoter at the transcription start site, but hypoacetylation found ∼1 kb upstream and downstream of intron 1. Treatment with histone deacetylase inhibitors resulted in a 2-fold increase in promoter activity on the *SMN2* locus (Kernochan et al., 2005). Through genome-wide methylation profiling, 10 CpG sites were identified as having significantly different methylation levels compared between healthy and diseased blood samples. Notably, differentially methylated CpG sites were found on the *ARHGAP22* gene, which produces a protein that converts Rho GTPases into an inactive state. Rho GTPases are heavily involved in regulating growth and branching of axons. Additionally, the cyclin dependent kinase two associated protein (CDK2AP1) locus, associated with G1/S cell cycle transition and apoptosis, was differentially methylated (Zheleznyakova et al., 2013; Virata et al., 2025). The SMN protein is thought to be involved in apoptosis, as it has been shown to protect against numerous apoptotic factors such as growth factor deprivation, camptothecin and PI 3-kinase inhibiton. The mechanism by which SMN is thought to inhibit apoptosis is by its regulatory action on calpain-mediated activation of procaspase-3. By blocking the activation of procaspase-3, the SMN protein inhibits active caspase-3 subunit formation, which is a key player in apoptotic cell death (Anderton et al., 2013).

SMN’s most notable role is assembling snRNPs, a complex necessary for the recognition of splice sites and removal of introns in pre-mRNA. An important structure in this process is the Cajal body (CB), where the final maturation of the snRNP occurs CBs are small, spherical structures located within the cell’s nucleus, usually near the nucleolus. Notably, MNs have a disproportionately high number of CBs. CBs are involved with the biogenesis of snRNPs and are rich in SMN and coilin (Andrade et al., 1991), a scaffolding protein that gives structural support to the CB. The CB’s close association with the nucleolus (Trinkle-Mulcahy and Sleeman, 2016), the site of rRNA production, suggests that this interaction is important for rRNA homeostasis and metabolism. Along the same path, CBs are thought to be more prominent in MNs because of the increased need for splicing and ribosome biogenesis to sustain their ability to constantly send and receive electrical signals (Lafarga et al., 2017; Girard et al., 2006). Low SMN protein reduces the number of CBs seen in MNs, and because of their close association with the nucleolus, ribosomes are also reduced. Another structure that closely interacts with CBs is Gems. Gems are “gemini of Cajal bodies,” small nuclear structures that colocalize with SMN and Gemin proteins (Feng et al., 2005). SMN may mediate the interaction between CBs and Gems, as RNAi-mediated reduction of SMN in HeLa PV cells results in the complete loss of Gems (Gubitz et al., 2004). Gemin proteins are important subunits of the SMN complex. The SMN complex facilitates the maturation of pre-snRNAs to snRNPs for spliceosome biogenesis (Liu et al., 1997).

**FIGURE 2:**
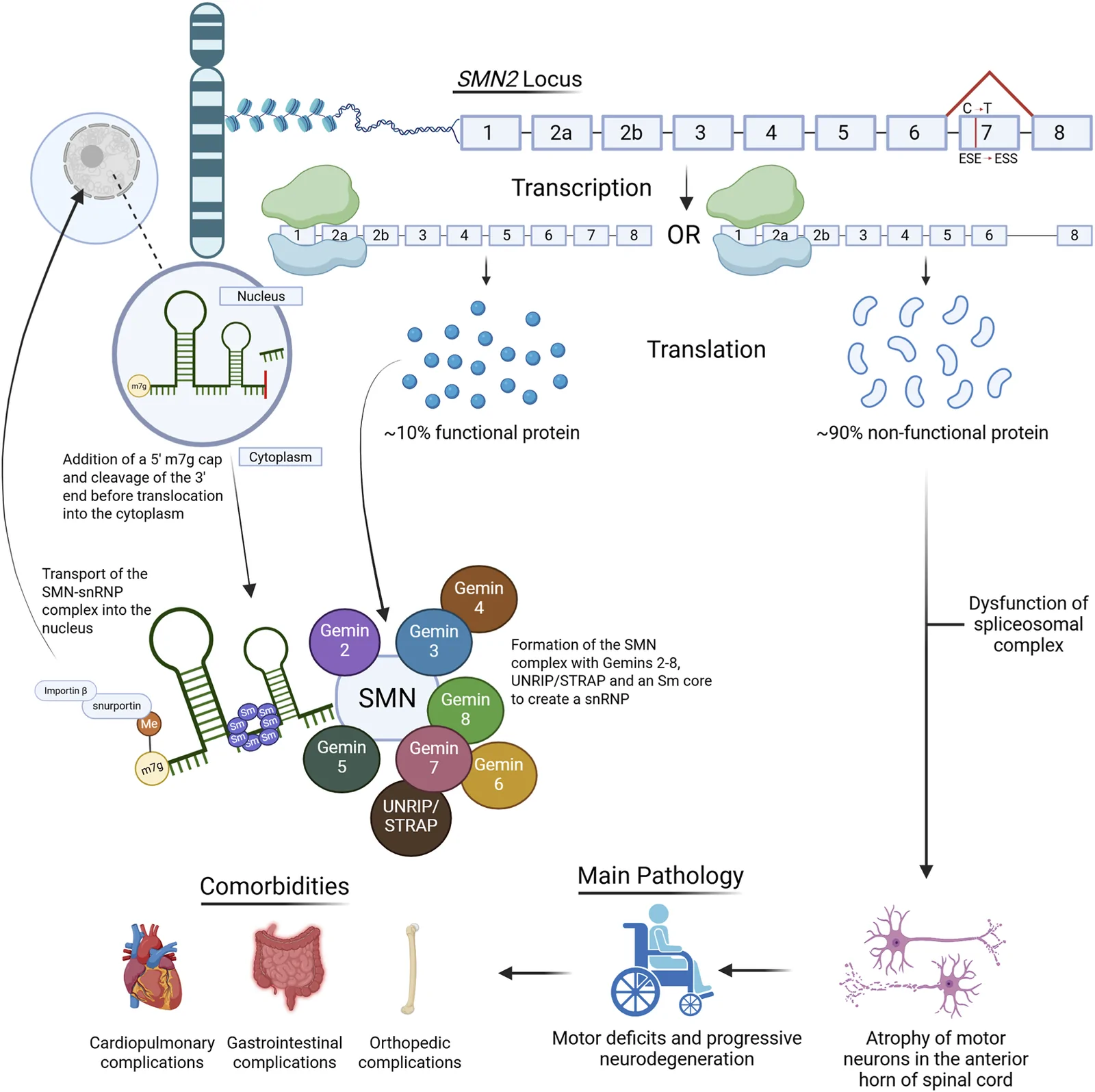
Formation of the SMN protein complex. A mutation within exon seven in *SMN2* results in 90% nonfunction SMN protein, causing dysfunction of the spliceosomal complex. Full-length SMN in the cytoplasm interacts with Gemins 2/3/5/7/8 directly, and Gemins 4/6 and UNRIP/STRAP indirectly. The SMN complex facilitates binding of the heptameric core of Sm proteins on the snRNA, to convert it to an snRNP. Snurportin binds to the hypermethylated m7 g cap, and importin *β* helps to facilitate translocation into the nucleus. Created in BioRender. Sexton, M. (2026) https://BioRender.com/xi7rxu6.

Production of snRNPs begins in the nucleus, with transcription of snRNAs. Before exporting into the cytoplasm, a 5′ 7-methylguanosine cap is added, and the 3′ end is cleaved. In the cytoplasm, the Tudor domain of SMN allows for the binding of C-terminal arginine and glycine-rich tails of Smith core (Sm) proteins (Chaytow et al., 2018). The Sm proteins are assembled in a heptameric ring around the snRNA by the SMN complex. The SMN complex is self-oligomerizing and composed of one–four subunits of SMN, along with Gemin proteins two to eight and UNRIP/STRAP (Fallini et al., 2011; Gruss et al., 2017). Gemins 2/3/5/7/8 interact directly with the SMN core, while Gemins 4/6 bind to Gemins 3/7, respectively. Following the assembly of the Sm protein onto the snRNA, the 7-methylguanosine cap is hypermethylated to allow for the binding of importin b and snurportin to mediate translocation into the nucleus (Figure 2). The SMN complex, along with the mature snRNP, localizes in CBs until they are mobilized for spliceosomal assembly (Burghes and Beattie, 2009).

### 3.1 Why is SMA a motor neuron selective disease?

Several hypotheses have been proposed regarding the mechanisms underlying MN’s selective vulnerability to reduced SMN levels. Most suggest that reduced SMN severely inhibits the assembly of spliceosomal complexes, disrupting genes critical for MN development. Indeed, SMN deficiency leads to a MN-specific decrease in snRNPs, resulting in splicing abnormalities in critical neuronal genes such as Stasimon (Lotti et al., 2012). Stasimon is regulated by SMN and is required for motor circuit transmission in *Drosophila* larvae and axon growth in zebrafish. Interestingly, Stasimon expression attenuates tumor suppressor p53 activity. Low levels of SMN lead to both p53 accumulation via a stabilization mechanism and p53 phosphorylation, resulting in cell death (Simon et al., 2017). Decreased Stasimon expression due to splicing defects caused by low SMN levels increases p53 activation, likely causing catastrophic MN death (Simon et al., 2019). Additionally, since SMN is differentially expressed at higher levels in MNs and not all snRNP complexes are severely inhibited by decreased SMN levels, it is possible that a deletion of *SMN1* would disproportionately affect the MN function compared to other tissues (National Center for Biotechnology Information (US), 1998).

Moreover, SMN interacts with numerous mRNA-binding proteins, including HuD, insulin-like growth factor 2 mRNA-binding protein 1 (IMP1), β-actin, and Ptbp2/PTBP2. HuD binds to SMN’s Tudor domain, a region commonly mutated in SMA patients. This mutation negatively affects SMN and HuD’s binding affinity but does not impact SMN’s localization within the axons. Knockdown of SMN in MNs resulted in decreased HuD protein and poly-A mRNA levels in the axon, suggesting a role for SMN in the transport and destination of poly-A mRNAs (Fallini et al., 2011). IMP1 interacts with SMN in granules, and the localization of IMP1 to MN axons is neuron-specific and depends on SMN concentration. β-actin is an important component of MN structural integrity by modulating axonal elongation. β-actin is found at high concentrations in the axons and growth cones of MNs; however, this distribution is abnormal in the presence of low SMN levels (Rossoll et al., 2003). Ptbp2/PTBP2 has also been reported to interact with SMN, specifically within cytosolic compartments of MNs. The absence of SMN reduces Ptbp2/PTBP2 expression in axons but not in the Soma, suggesting that SMN may play a role in the spatial distribution of protein synthesis (Salehi et al., 2024).

Another theory that explains the MN-specific pathology of SMA is that SMN plays an important role in axon outgrowth, neuronal development, and the integrity of neuromuscular junctions (NMJs). NMJs are a major focus of SMA research, as the loss of connection with muscular tissue contributes greatly to the symptoms experienced by SMA patients. One of the first indications for denervation is the breakdown of the NMJs, occurring before symptoms even begin (Zhang et al., 2013; Lee et al., 2011; Kariya et al., 2008). A transcriptome analysis of MNs and white matter from lumbar sections of SMA mice shows changes in the expression of around 300 genes, including neurotrophic factors Igf1/2 and sensory-motor circuit protein Etv1. These expression changes are seen before symptom onset (Zhang et al., 2013), suggesting that this aberrant function could be prevented. Breakdown of NMJs due to low SMN has also been associated with reduced endocytosis and exocytosis at the synapse. In a severe SMA mouse model, abnormal synaptic transmission was observed due to decreased synaptic vesicles and a delay in the maturation of fetal acetylcholine receptors to adult receptors (Kong et al., 2009). Furthermore, terminal arborization is significantly impacted, along with neurofilament aggregation in pre-synaptic terminals (Kariya et al., 2008). Endocytic pathways are also negatively affected, with fewer docked presynaptic vesicles, irregular cisternae, and defects in non-neuronal tissues (Dimitriadi et al., 2016). Hyperexcitability has also been proven to interfere with neurotransmission in patient MNs. These MNs have a hyperpolarized threshold and require a larger action potential, contributing to impaired neurotransmission (Liu et al., 2015; Gogliotti et al., 2012).

It is evident that SMNs role in RNA metabolism and axonal integrity is closely intertwined, as nearly all of SMN’s interactions with mRNA binding proteins affect axon growth and function. SMN is likely a major facilitator for the transport and localization of proteins critical to axon and NMJ maturation and continued vitality. It will be necessary to investigate these interactions further and differentiate between direct and indirect targets for treatments. Additionally, analysis of downstream pathways, especially those implicated in neurotransmission and muscular function, may reveal further insight into SMN’s specific function within MNs.

## 4 Disease modifying therapies and multidisciplinary care for SMA

Before DMTs were discovered, the diagnosis of SMA was devastating. Children diagnosed with Type I had a life expectancy of ∼2 years. Patients with Types II-IV had longer lifespans, but progressive loss of motor abilities made daily life more difficult, often requiring the help of a full-time caregiver. Currently, there are three FDA-approved treatments for SMA (Table 1). While significantly improving patient quality of life, these treatments are not curative and only slow the progression of the disease.

**TABLE 1:** Characterization of FDA-approved DMTs.

Treatment	Mode of action	Route of administration and dosing frequency	Qualification for treatment	Key limitations
Nusinersen *(Spinraza)*	Antisense oligonucleotide*, SMN2* splicing modifier	Intrathecal injection, every 4 months following the loading phase	All ages	Does not cross the blood brain barrier (Day et al., 2022) Requires frequent intrathecal injections, which may not work for all patients
Onasemnogene abeparvovec *(Zolgensma)*	*SMN1* gene replacement via AAV9 vector	One intravenous injection	Less than 2 years	Excludes late-onset patients who did not receive genetic screening
Risdiplam *(Evrysdi*)	*SMN2* splicing modifier	Orally, every day	Older than 2 months	Nonadherence to daily treatment can impact treatment effectiveness (Patel et al., 2024)

### 4.1 Nusinersen

Nusinersen is an antisense oligonucleotide (ASO) that binds to a specific site within the *SMN2* pre-mRNA to encourage the inclusion of exon seven in the mature transcript. In the early stages of its development, researchers demonstrated that an ASO designed to target *SMN2* RNA could promote inclusion of exon seven *in vitro* (Lim and Hertel, 2001). Further investigation into sequence elements regulating the alternative splicing of *SMN2* revealed an intronic splicing silencer, ISS-N1 (Hua et al., 2008). The development of an ASO targeting ISS-N1 resulted in a significant increase in exon seven inclusion in the mature mRNA transcripts (avg of 88% at 10 and 20 µg doses) in an SMA Type III mouse model (Hua et al., 2010). The first clinical trials began in 2011. In 2016, Biogen filed for accelerated FDA approval following a Phase 3 clinical trial (ENDEAR) that showed significant improvement in infants with SMA Type 1. Importantly, they showed that nusinersen was most effective when administered earlier (Qiu et al., 2022). A table outlining the important phases of Nusinersen is presented in Table 2. Notably, data from the DEVOTE study on a higher-dose regimen of nusinersen in both the loading and maintenance phases was approved by the FDA in early 2026. The safety profile was generally consistent with that of the lower-dose nusinersen, which is supported by 10+ years of clinical data (Biogen, 2026).

**TABLE 2:** Summary of previous clinical trials pivotal to the advancement of nusinersen.

Trial name	Phase	Population	Specifications
CS1 NCT01494701 (Haché et al., 2016)	Phase 1	2–15 years	Exclusion of patients needing ventilatory support
CS2 NCT01703988 (Darras et al., 2019)	Phase 1/2	infants – 15 years	Exclusion of patients needing ventilatory support
CS10 NCT01780246 (ClinicalTrials.gov, 2021a)	Extension of CS1	​	​
CS12 NCT02052791 (ClinicalTrials.gov, 2021b)	Extension of CS2 and CS10	​	​
CS3A NCT01839656 (Finkel et al., 2016)	Phase 2	21 days-7 months	No *SMN2* copy number restriction, symptoms at or before 6 months
ENDEAR NCT02193074 (ClinicalTrials.gov, 2021c)	Phase 3	21 days-7 months	2 *SMN2* copies, symptoms at or before 6 months
CHERISH NCT02292537 (Mercuri et al., 2018b)	Phase 3	2–12 years	Symptoms after 6 months, sits independently, excluded patients with gastric tube feeding
NURTURE NCT02386553 (Crawford et al., 2023)	Phase 2	<6 weeks	2–3 copies of *SMN2*
EMBRACE NCT02462759 (Acsadi et al., 2021)	Phase 2	Infants/children	Patients excluded from CHERISH or ENDEAR
SHINE NCT02594124 (Kokaliaris et al., 2024)	Extension	​	Durability/safety follow-up from CS1, CS2, CS10, CS12
DEVOTE NCT04089566 (Finkel et al., 2026)	Phase 2/3	All populations	Higher dose
ONWARD NCT04729907 (ClinicalTrials.gov, 2026a)	Extension of DEVOTE	​	​
ASCEND NCT05067790 (ClinicalTrials.gov, 2026b)	Phase 3b	Teenagers-adults	Higher dose of nusinersen with previous treatment with risdiplam

### 4.2 Onasemnogene abeparvovec

Onasemnogene abeparvovec is a non-replicating adeno-associated virus serotype 9 (AAV9) vector that can deliver the full-length *SMN1* gene. The delivered *SMN1* gene is under the control of the cytomegalovirus enhancer/chicken-*β*-actin hybrid promoter, and the AAV9 inverted terminal repeat is modified to enhance annealing of the transgene (Foust et al., 2008; Ogbonmide et al., 2023). Onasemnogene abeparvovec is delivered systemically and can cross the blood-brain barrier to reach spinal MNs. It is a one-time, intravenous injection that can only be administered to patients under 2 years old. A meta-analysis conducted 4 years after the drug’s initial release shows mostly positive results, especially in patients treated earlier in life. In most studies, patients improved their motor milestone scores, and patient outcomes were more successful than with nusinersen (Ogbonmide et al., 2023). A table outlining the important trials in the development of onasemnogene abeparvovec is listed in Table 3. A biodistribution study of onasemnogene abeparvovec in post-mortem SMA patient tissue showed the vector genome in all analyzed tissues, with the thymus having least, and the liver having the most. Immunohistochemistry revealed SMN expression throughout the CNS, including the spinal cord, motor cortex and the brainstem. Additionally, SMN was expressed in peripheral tissues, including skeletal muscle, heart, lung, and stomach (Thomsen, et al., 2021).

**TABLE 3:** Summary of previous clinical trials pivotal to the advancement of onasemnogene abeparvovec.

Trial name	Phase	Population	Specifications
START NCT02122952 (Mendell et al., 2017)	Phase 1	<7 months	SMA Type 1
START-LT NCT03421977 (Waldrop et al., 2026)	Extension	​	Long term follow up study from patients enrolled in START
STR1VE-US NCT03306277 (Day et al., 2021)	Phase 3	<6 months	SMA Type 1, residing in the United Staes, exclusion of those requiring ventilatory support for >7 days and failure of swallowing tests
STR1VE-EU NCT03461289 (Mercuri et al., 2021)	Phase 3	<6 months	SMA Type 1, inclusion of patients with a more severe disease baseline, like those requiring 12+ h of ventilatory support or nutritional support
SPR1NT NCT03505099 (Strauss et al., 2022)	Phase 3	<6 weeks	Presymptomatic, 2 or 3 copies of *SMN2*
LT-002 NCT04042025 (ClinicalTrials.gov, 2025)	Extension, follow up	​	Any patient who previously participated in an onasemnogene abeparvovec clinical trial
STRONG NCT03381729 (Finkel et al., 2023)	Phase 1	6-<24 months or 24-<60 months	Sitting, non-ambulatory
STEER NCT05089656 (Proud et al., 2025)	Phase 3	2 years - <18 years	Treatment-naïve, sitters but never walkers
STRENGTH NCT05386680 (Kwon et al., 2026)	Phase 3b	2-<18 years	Patients who discontinued nusinersen or risdiplam

### 4.3 Risdiplam

Risdiplam is the most recent FDA-approved treatment for SMA. Like nusinersen, it is an *SMN2* splicing modifier. Risdiplam differs from the other treatments as it is an organic molecule, C_22_H_23_N_7_O, or 7-(4,7-diazaspiro [2.5]octan-7-yl)-2-(2,8-dimethylimidazo [1, 2-b]pyridazin-6-yl)pyrido [1, 2-a]pyrimidin-4-one (Balińska et al., 2025). Several analogs of risdiplam exist, and many were found to interact with GA-rich regions in single stranded RNA. It is proposed that the binding of risdiplam to these GA-rich regions recruits far upstream element binding protein one and KH-type splicing regulatory protein, preventing these proteins from reaching their intended target (Ottesen et al., 2023). The analog of risdiplam marketed as Evrysdi works by stabilizing the complex between the 5′ splice site on exon seven and the U1 small nuclear ribonucleoprotein, making the splice site stronger and less likely to degrade. Risdiplam is highly specific to the 5′ splice site of SMN2 exon 7, likely due to its ability to also selectively bind to the exonic splicing enhancer motif (Ratni et al., 2021). Fortunately, risdiplam can cross the blood brain barrier as it does not bind to multi-drug resistant protein 1, and therefore cannot be effluxed out (Kakazu, et al., 2021). Risdiplam is unique in that it is taken orally, either as a liquid or a pill and requires no hospital visits for treatment. A recent case study investigated prenatal therapy of risdiplam on a confirmed SMA type I fetus by administering the drug orally to the mother starting at 32 weeks, 5 days gestation until birth. The infant was given risdiplam beginning on postnatal day 11. At 30 months of age, no manifestations of SMA were present. Although additional trials are needed, the results of this case study reveal a promising early intervention treatment strategy (Finkel, et al., 2025a). In a matching-adjusted indirect comparison, SMA Type 1 patients who were treated with risdiplam (FIREFISH) resulted in a 78% reduction rate of death and a 45% higher Hammersmith Infant Neurological Examination 2 (HINE-2) score compared to Type 1 patients treated with nusinersen (ENDEAR/SHINE) (Kokaliaris et al., 2024). A table outlining the pivotal clinical trials of risdiplam is shown in Table 4. A recent study examining the transcriptome wide effects of varying doses of risdiplam in SMA fibroblasts reveal valuable information both on personalized dosing for patients and novel motifs found to impact splicing of *SMN2* exon 7. In the high dose group (1 µM) 3,670 genes were differentially expressed with a >2 fold change, whereas in the low dose group (50 nM), only one differentially expressed gene had a >2 fold change. Interestingly, genes with multiple transcripts were preferentially affected within the high dose group. Aberrant expression of genes involved in RNA stability, tumor suppression and DNA replications were present in the high dose group. Using previously reported regions affecting exon seven splicing and the effects of risdiplam on the hybrid minigenes created in this study, the authors proposed that GG residues in positions 26 and 27, and AC residues at positions 36 and 37 are vital to the mechanism of exon seven inclusion by risdiplam (Ottesen et al., 2023).

**TABLE 4:** Summary of previous clinical trials pivotal to the advancement of risdiplam.

Trial name	Phase	Population	Specifications
FIREFISH NCT02913482 (Masson et al., 2022)	2	Infants	Type 1 SMA, part 1 was an exploratory dose finding, and part 2 was confirmation of dose
JEWELFISH NCT03032172 (Chiriboga et al., 2024)	Phase 2	All populations	Patients who previously participated in a clinical trial of nusinersen, onasemnogene abeparvovec, or olesoxime
SUNFISH NCT02908685 (Mercuri et al., 2022)	Phase 2	2–25 years	Type 2 and 3 SMA. Part 1 was exploratory dose finding, and part 2 was confirmation of dose
RAINBOWFISH NCT03779334 (Finkel et al., 2025b)	Phase 2	<6 weeks	Presymptomatic
MANATEE NCT05115110 (ClinicalTrials.gov, 2026c)	Phase 2/3	2–25 years	Patients receive a combination of risdiplam and RO7204239, an investigational anti-myostatin antibody. Part 1 is dose finding in ambulant (2–10 years) and non-ambulant (5–10 years) patients. Part 2 focused on ambulant patients 2–25 years

Each of the three available DMTs has completely changed patients’ outcomes, quality of life, and life expectancy. Not only do they stop the progression of the disease, but they also provide patients with a variety of different treatment options that best suit their personal needs and lifestyle. Nusinersen requires invasive, intrathecal injections every 4 months, which may not be the best option for patients with scoliosis or who have difficulty accessing treatment facilities. It has the strongest evidence for improvement in pre-symptomatic patients, with moderate side effects. Onasemnogene abeparvovec is only available for patients under 2 years old, which limits the treatment options for adults living with SMA. It showed the highest early motor gains but is associated with the most adverse effects. Risdiplam is taken orally, which may be difficult for patients with swallowing difficulties. It has the fewest side effects, and 80% of patients demonstrated an increase in motor ability (Rathod et al., 2025).

CureSMA has one of the largest databases encompassing patients of SMA and their caregivers, representing about 70% of the current U.S. SMA population. Using surveys and patient data, they estimate the survival and characteristics of the SMA population. These surveys provide important data that help researchers identify the most significant challenges faced by patients and their caregivers. The relevance of these data has grown substantially with the introduction of DMTs, which have improved survival and highlighted previously underrecognized challenges that warrant ongoing investigation and intervention. Indeed, since the dawn of the DMT era, the mortality rate of SMA has dropped by nearly 80%. However, 36% of patients report needing to use some form of breathing support, and 89% of adults report that reaching new motor milestones is their greatest unmet need (Cure, Cure SMA, 2025). The progress made thus far represents only the beginning of a new era in SMA care. Continued improvements in patient outcomes will likely depend on the development of combination treatment strategies that build upon the benefits of existing DMTs while addressing residual disease burden and emerging unmet needs.

### 4.4 Multidisciplinary care

Multidisciplinary care for SMA patients is critical to reducing the disease burden. Each case of SMA is unique and requires a team of specialists working to improve the quality of life for patients. Comprehensive care management can include rehabilitation of motor, respiratory, and swallowing functions. Functional motor therapy for patients most commonly involves physical therapy, stretching exercises and assistive seating systems. Assistive seating helps patients move around, in the case of an electric wheelchair, and to control posture and prevent hip dislocations. Head and neck braces are also used as a supportive measure (Boulay, et al., 2020). Respiratory rehabilitation is critical to patient safety and comfort. A non-invasive positive pressure ventilation mask can deliver oxygen to patients and can be utilized in the home. Respiratory muscles can be strengthened through exercise, and airway clearance strategies like manual cough assistance and postural drainage can be implemented (Song and Ke, 2025). Swallowing difficulties are recommended to be assessed by videofluoroscopic swallow imaging, but access to specialized equipment is limited, and requires trained personnel (Winnicka, et al., 2025). A nasogastric feeding tube can be used when patients regress in motor control of their tongue and neck muscles (Gandolfi et al., 2026). As a result of eating difficulties, some patients may experience nutritional deficiencies and metabolic dysfunction (Blauciak et al., 2025)While SMA patients are the focus of this review, it would be remiss to not acknowledge the critical role caregivers and families play in the life of a patient with SMA. The progressive nature of SMA requires heavy involvement of caregivers in daily activities, which, over time, can cause physical, emotional, and financial stress. In many cases, caregivers are responsible for transportation to and from office visits, making or aiding in treatment decisions, and ensuring the highest quality of life (Song and Ke, 2025). A study found that poor caregiver mental health predicted a higher patient mortality rate in patients with neurodegenerative diseases (Lwi, et al., 2017), highlighting the critical need for both patient and caregiver advocacy. Advocacy groups have been instrumental in improving diagnosis and care through newborn screening programs and the establishment of standard care guidelines. They have also raised awareness of the critical financial and social burdens experienced by caregivers and patients with SMA to policymakers to hopefully influence change within local and national communities (SMA Foundation, 2026).

## 5 Alternative targets and therapies

Advances in SMA therapeutics promise to bring a new generation of treatments and the identification of novel drug targets. iPSCs have emerged as valuable tools for *in vitro* modeling of SMA and hold considerable promise as a regenerative treatment strategy. Other types of stem cells have also been investigated for their proliferative and therapeutic potential. In addition, non-stem cell therapies can target specific genes or pathways that could help manage the comorbidities experienced by SMA patients.

### 5.1 iPSCs

iPSCs have been a useful tool for developing new screening strategies, biomarker assessments, and therapies for SMA. iPSCs can be easily obtained from patients’ skin fibroblasts in a less invasive manner and reprogrammed to mimic the patient’s MN cells (Chen et al., 2025). Due to the early onset of SMA, it can be difficult to obtain viable neural stem cells (NSCs) from patients (Frattini et al., 2015), making iPSC lines valuable for new drug development. iPSC transplantation can recapitulate MN structure and function. NSCs derived from iPSCs have been shown to have a therapeutic effect in a subset of SMA, spinal muscular atrophy with respiratory distress type 1 (SMARD1). Transplantation into the spinal cord resulted in proper engraftment within the damaged environment and, via kinase inhibition, the secretion of neurotrophic factors that helped restore native cells (Simone et al., 2014). Another study using iPSCs that were pre-treated with neurotrophic factors and transplanted into the cerebrospinal fluid of a SMARD1 mouse model also showed positive effects, including improved NMJ function and muscle fiber morphology (Forotti et al., 2019).

Studies using iPSC lines have identified numerous molecules, pathways, and proteins that are affected by SMN deficiency. iPSC lines generated from a Type 1 SMA patient had increased Fas ligand-mediated apoptosis and upregulated caspase 8/3 activation. Inhibition of these pathways could potentially be a therapeutic target (Sareen et al., 2012). Cardiomyocytes are also implicated in conditions of low SMN levels. Patient-derived iPSC lines reduced the function of cardiomyocytes through dysregulation of calcium reuptake via SERCA2 disruption, which was improved with SMN restoration (Khayrullina et al., 2020). Moreover, heart failure markers such as creatine kinase and brain natriuretic peptide increase in a low SMN environment (Khayrullina, 2021). In addition, there have been many reports of patient astrocytes that are glial fibrillary acidic protein (GFAP) positive, indicating neurotoxicity. Inhibition of the Notch signaling pathway, which is important for astrocyte proliferation, reduced motor deficits in SMA mice (Ohuchi et al., 2019). Patient-derived iPSC lines have also been used to help develop a connectivity map (CMAP), coined CMAP_neuro-_ by differentiation into NGN2 cells and exposing them to 360 neurological irritants to measure transcriptional changes for better prediction of how a compound will affect SMA (Giadone et al., 2026).

### 5.2 Non-iPSC cell therapy

Non-iPSC cell lines have also been pivotal in SMA research. Prenatal injection *in utero* of human amniotic stem cells into SMA mice resulted in a 93.75% survival rate at birth, good motor function and a significantly higher number of myocytes, neurons and innervated receptors (Shaw et al., 2021). Neural stem cell transplantation into SMA mice showed proper migration into the parenchyma, MN differentiation, increased lifespan, and positive changes in RNA metabolism (Corti et al., 2008). Fibrin matrices embedded with NSCs, and growth factors were successfully engrafted into a spinal cord injury model and were able to differentiate into neurons that connected with the native tissue. Axonal growth and synapse formation were observed, suggesting cell scaffolding could promote stem cell incorporation following transplantation (Lu et al., 2012). However, non-iPSC-derived NSCs are difficult to obtain and expand *in vitro* to the degree needed for transplantation (Nam et al., 2015).

### 5.3 Other treatment strategies

Non-cell therapies have also been shown to be beneficial in treating SMA pathology. The anti-epileptic drug levetiracetam was used to treat MNs from patient-derived iPSCs and had an overall neuroprotective effect. It encouraged neurite elongation, the expression of cleaved-caspase three was decreased, and mitochondrial function was improved (Ando et al., 2019). Edaravone, a common drug used to treat ALS, is a free radical scavenger. Treatment of patient iPSCs with edaravone inhibited the generation of reactive oxygen species and prevented oxidative stress-induced apoptosis in MNs (Cha and Kim, 2022). Antioxidant curcumin, obtained from turmeric, has also been shown to improve disease phenotype *in vitro* by activating nuclear factor erythroid 2-related factor 2 (NRF2), a regulator of cell resistance to antioxidants. This treatment did not affect the expression of NSC markers and increased the amount of SMN protein expression, but did not restore functionality to the wild type (Adami et al., 2024). A Drosophila-based drug repositioning model identified haloperidol, an antipsychotic drug, as a potential therapeutic agent by its ability to increase *SMN2* expression through alternative mechanisms like inclusion of exon 8 (Menduti et al., 2026). An alternative, non-drug electrical stimulation strategy has been tested in a cohort of three adults with SMA. Epidural electrodes were implanted along the lumbosacral spinal cord, aimed to target sensory MN in the legs. Motor abilities in patients improved significantly over 4 weeks, with 2 h per day of treatment resulting in improvements in strength, a 40% increase in mean step length, and a +26 min increase in an endurance walking test. Effects remained even when the stimulator was off (Prat-Ortega et al., 2025).

Alternative forms of gene therapies are also being researched. A one-time base editing technique was developed targeting different *SMN2* regulatory regions, which resulted in an average of 87% permanent conversion of the C to T point mutation in SMNΔ7mice. Both *in vivo* and *in vitro* analysis yielded no off-target effects, and effects are not transient as nusinersen and risdiplam are (Arbab et al., 2023). Another study used adenine base editing to target the C to T point mutation and achieved up to 90% successful editing in patient fibroblast cells using a broad-targeting enzyme SpRY. Once tested in an SMNΔ7 model, they observed a 4% base edit in the spinal cord and a 6% base edit in the brain, consistent with other studies using similar methods. *SMN1* transcript levels were increased by a two to 4-fold increase (Alves et al., 2024). A second-generation AAV9 vector, identical to onasemnogene abeparvovec, was developed to address safety concerns observed in patients following treatment. The newly designed vector used an AAV9 construct that expressed a codon-optimized *SMN1* gene under the control of an endogenous *SMN1* promoter. When directly compared with onasemnogene abeparvovec, transaminase levels remained stable, suggesting hepatic safety and a lower dose to achieve therapeutic efficacy (Xie et al., 2024). The CRISPR-Cas9 system also holds considerable potential as a therapeutic strategy for SMA, especially following the FDA approval of Casgevy for the treatment of sickle cell disease (Singh et al., 2024). CRISPR-Cas9 is already being investigated for its potential therapeutic value in SMA. A prime editing strategy was used to target the ISS-N1 sequence by a Cas9 nickase in SMA patient iPSCs. A pegRNA with the edited sequence was reverse transcribed into the genome, causing a disruption to the silencer sequence and improving full-length *SMN2* production (Zhou et al., 2022). Li et al. used Cas9 and guide-sgRNAs designed to target the intronic splicing silencer elements on *SMN2* to promote exon seven inclusion. This disruption resulted in full length SMN protein expression *in vitro*, and 62.8% of severe SMA mice survived over 400 days after germline correction with the treatment (Li et al., 2019). Another study utilized Cpf1, a simpler more precise endonuclease to convert SMN2 into SMN1 by converting the mutated T back to a C. Using Cpf1 is advantageous, because it does not require G-rich PAM sequences, In this study, introducing a mutation in the PAM sequence near the target site did not allow for additional cutting by the endonuclease, effectivly creating a blocking mutation (Zhou et al., 2018).

SMA is a complex disease where multiple biological mechanisms contribute to and exacerbate disease progression. As our understanding of the effects of chronic deficiency of the SMN protein grows, new therapeutic targets are being identified as potential disease modifiers. miRNAs have been identified as stressors for SMA, as they can post-transcriptionally silence multiple mRNA transcripts. miR-9, a neruon-specific miRNA, showed significant downregulation in the spinal cord of SMA mice, but increased threefold in skeletal muscles. miR-132, expressed in both the nervous system and peripheral tissues, decreased in spinal cord tissue, but significantly increased in skeletal muscle (Catapano et al., 2016). Correction of the abnormality using a morpholino antisense oligomer restored levels to basal levels. miR-23a has also been suggested as a disease modifier, as it can suppress Atrogin1 and MuRF1, both of which play a role in muscle atrophy. mir-23a is downregulated in SMA patient iSPCs. Delivery through an AAV9 vector into a mouse model increased MN Soma size and increased the number of innervated synapses in the surrounding muscle (Kaifer et al., 2019). Targeting the NMJ is also a potential therapeutic target. Munc13-1 is a pivotal molecule in the docking of synaptic vesicles and synaptic plasticity. The axonal localization of Munc13-1 is dependent on SMN, resulting in low translational efficiency. Overexpression of a Munc13-1 construct in SMN knockout MNs resulted in improved neurotransmitter release and enhanced excitability (Moradi et al., 2025). Connexin 43 is a gap junction protein that is heavily expressed in astrocytes and upregulated in later-onset SMA. Overexpression results in aberrant Ca^2+^ homeostasis but targeting the hemichannel blocker Gap27 recapitulates glutamate release and Ca^2+^ response in mouse and human astrocytes (Salmanian et al., 2025).

Apitegromab is an investigational myostatin inhibitor that is currently being clinically investigated as a combinatorial treatment with DMTs. It is a human monoclonal antibody that inhibits myostatin’s negative regulatory control over skeletal muscle growth. IV infusions have been tested in patients older than 2 years old also taking nusinersen (TOPAZ, NCT03921528). Patients with later onset SMA experienced improved motor function, with n mean increase of HFMSE score of 0.6 points. No hypersensitivity reactions were recorded, and all patients tested negative for anti-apitegromab antibodies (Crawford et al., 2024).

## 6 Discussion

Much effort has been put into investigating SMA pathology, mechanisms, and therapeutic targets. Decades of research have resulted in 3 FDA-approved therapies that have significantly improved patient outcomes and life expectancy. Before the availability of these commercially available treatments, a diagnosis of SMA Type 1 was devasting. In just a decade, we have gone from palliative care until death to achieving motor milestones that were never thought possible. There is still much work to do to ensure all facets of SMA disease pathology are addressed. SMA is a complex, multi-organ disease that will likely require a multi-factorial treatment plan. A combination of available gene therapies and additional treatments could address the shortcomings still being experienced by patients, including muscle weakness and respiratory support needs. Most importantly, we should aim to improve patient quality of life and ensure all ages of SMA patients and their specific concerns are represented and considered in future treatments and therapies.
